# On the Role of Personal Semantic Memory and Temporal Distance in Episodic Future Thinking: The TEDIFT Model

**DOI:** 10.3389/fnhum.2016.00385

**Published:** 2016-07-29

**Authors:** Valentina La Corte, Pascale Piolino

**Affiliations:** ^1^Institute of Psychology, University Paris DescartesParis, France; ^2^Memory and Cognition Laboratory, Center of Psychiatry and Neurosciences, Institut National de la Santé et de la Recherche Médicale UMR 894Paris, France; ^3^Initiatives d'excellence (IDEX) ‘Dynamique du Vieillir’, Université Paris DiderotParis, France; ^4^University Institute of FranceParis, France

**Keywords:** time travel, episodic prospection, future thinking, personal semantic memory, autobiographical memory, Alzheimer's disease, semantic dementia, hippocampus

In the last two decades, the study of memory processes has been expanded to encompass the processes involved in foreseeing events in the future dimension. The mental time travel is a mental ability, which allows individuals not only to go back in time but also to foresee specific future events (Suddendorf and Corballis, 1997).

The ability to project the self forward in time to pre-experience personal events is referred to as *episodic future thinking (EFT)* (Atance and O'Neill, 2001). This capacity has a strong adaptive and social value (Tulving, 2002; D'Argembeau and Van der Linden, 2012). While evidence is accruing regarding the existence of semantic general forms of prospection (Klein, 2013; Irish et al., 2016), it seems relevant to envisage the role of personal semantic representations in prospection. Here we focus on the impact of personal semantics depending on temporal distance in episodic forms of foresight. In what follows we (I) give a brief overview of different EFT accounts, (II) discuss the role of personal semantics in EFT, (III) propose a neurocognitive model, in which the role of personal semantic information in EFT is defined as a function of temporal distance, (IV) speculate about temporally graded EFT deficits in Alzheimer's disease and semantic dementia.

## Different theoretical accounts in episodic future-oriented thoughts

The most part of the recent theoretical accounts proposed to shed light on neurocognitive mechanisms underlying episodic prospection have been focused on the involvement of episodic memory system in the construction and elaboration of specific and vivid future thoughts. In this line, autonoetic consciousness, linked to episodic memory, seems to be crucial to re-experience past events and pre-experience future ones, that ground our sense of temporal continuity and personal identity (Tulving, 2002; Klein, 2013).

Striking evidence of the relation between episodic memory and EFT comes from neuroimaging studies providing evidence of brain similarities including hippocampus (Addis et al., 2007; Szpunar et al., 2007; Spreng and Grady, 2010; Viard et al., 2011), and neuropsychological studies conducted on Alzheimer's disease (AD) patients, which have highlighted EFT deficits, strongly correlated with an episodic memory deficit in this pathology (Irish et al., 2012; Irish and Piolino, 2015). These findings corroborate the *constructive episodic simulation hypothesis*, which states that the extraction of episodic details from past memories and their flexible recombination is fundamental to the successful generation of novel future scenarios (Schacter and Addis, 2007). More recently, neuropsychological investigations in semantic dementia patients (Duval et al., 2012; Irish et al., 2012) have revealed the importance of general semantic memory in episodic forms of future thoughts giving rise to the *semantic scaffolding hypothesis*. Accordingly semantic memory may have a pivotal role for episodic prospection (Irish and Piguet, 2013). Otherwise Szpunar et al. (2014) have proposed a new taxonomy of prospection including various forms of future thinking which vary according a semantic-episodic continuum.

## The personal semantic information and its role in episodic future thoughts

Despite increasing interest in general semantic knowledge in cognitive prospection, the specific role of personal semantics (PS) in EFT remains largely unexplored.

The main feature of PS is that it concerns semantic aspects of autobiographical memory (Conway and Pleydell-Pearce, 2000), and is highly personal (like episodic memory), yet at the same time devoid of any sense of recollection and detached from its context of acquisition (like semantic memory; Renoult et al., 2012).

PS is a complex system, which consists of different kinds of knowledge about the self, including personality traits and roles, personal beliefs and generic autobiographical facts (Martinelli et al., 2013; Grilli and Verfaellie, 2014). However different forms of PS are considered to vary in the extend to which they are mostly concept-based (i.e., traits, roles and beliefs considered highly conceptual and abstracts) or somewhat experience-near (i.e., some autobiographical facts associated with spatial-temporal and perceptual details like generic personal event memories; Grilli and Verfaellie, 2014).

Different levels of specificity exist because most memories undergo various modifications over time ensuring a transition from episodic to semantic memory by a process of abstraction, i.e., a semanticization process (Cermak, 1984; Piolino et al., 2009). At the neural level, studies have shown that the semanticization of autobiographical memories over the time is accompanied by a disengagement of hippocampal activity in memory retrieval (Moscovitch et al., 2005), but that some remote memories (highly self-relevant and emotional) remain episodic in nature and are dependent of hippocampal activity (Viard et al., 2007). Otherwise, the role of PS is crucial in the reconstruction of episodic autobiographical memory (EAM) as most abstract personal information gives access to most specific ones allowing the revival of episodic memories (Conway, 2005; Piolino et al., 2010). PS and EAM are broadly involved in maintaining the sense of identity and personal continuity over time (Prebble et al., 2012). Within this framework, EAM is related to the subjective aspect of sense of self in time (“I feel myself” in time, James, 1890), while PS is related to the conceptual aspect (“I know myself,” “Me,” James, 1890; Prebble et al., 2012; Abram et al., 2014). Thus, the individual's capacity to mental time travel involves both phenomenological continuity as well as semantic continuity which are necessary to imagine and predict future personal events. Accordingly, Klein (2013) states that these different types of self-knowledge are involved in personal prospection. In this line, Coste et al. (2012) demonstrated that mechanisms of construction of episodic events via PS are similarly involved both in the past and the future. However, the specific role of the PS as a function of different temporal distances of prospection remains still unclear.

## Episodic future thoughts, personal semantic memory and temporal distance (TEDIFT model, “*temporal distance in future thinking*”)

**Figure 1:**
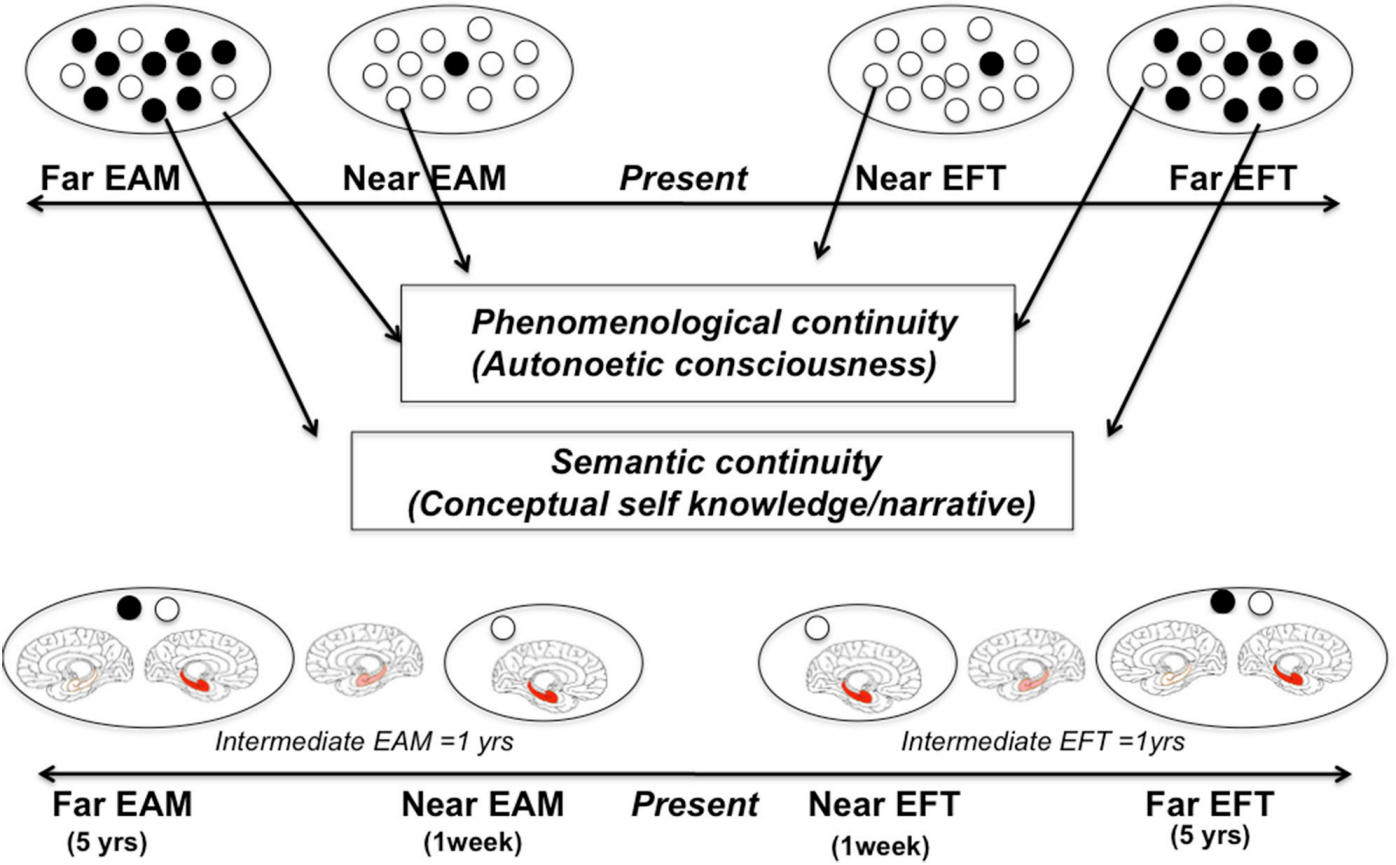
**Model of the role of personal semantic memory (PSM) in episodic future thinking (EFT) as a function of temporal distance**. Top: Black dots indicate personal semantic (PS) representations in past and future episodic thoughts whereas white dots are related to episodic autobiographical past (EAM) and future (EFT) representations. Temporal distances: near (1 week), intermediate (1 year), distant (in 5 years) past and future temporalities. We hypothesize that the involvement of PS in personal thoughts increases with the temporal distance both in the past and the future. In this context, (1) different kinds of personal prospection exist: a vivid form concerns personal prospection (mostly in near temporal contexts), based on phenomenological continuity (related to autonoetic consciousness), and a more general form based on PS (semantic continuity), which is necessary to envisage more distant temporal contexts; (2) PS is gradually more involved in constructing EFT as a function of temporal distance; (3) distant forms of EFT exist as far as self-relevance and emotion are concerned. Bottom: Representation of gradual decrease of hippocampal activity as a function of temporal distance. In the near forms of personal future thoughts the hippocampal activity is deeply involved, whereas a progressive decrease in such activity associated to a progressive increase in neocortical activity is observed in intermediate and distant forms of personal future thoughts. Nevertheless, the hippocampus is involved in EFT regardless of temporal distance as far as self-relevance and emotion are concerned. The model proposed here can be considered as an extension to the future dimension of the consolidation model proposed by Nadel and Moscovitch (1997).

Some studies have shown that the sense of pre-experiencing the future is modulated by temporal distance (e.g., comparing next week, next year, and in 5 years), and by the self-relevance attributed to the specific event (D'Argembeau and Van der Linden, 2004; Abram et al., 2014; Murphy et al., 2015). For instance, the more distant the events are from the present, the less mental visual imagery and sense of pre-experiencing they carried. This indicates that distant future events are mentally represented in a more abstract way than closer future events in accordance with the *construal level theory* that states that the temporal distance influences the way individuals construct future events, represented in terms of abstract or concrete features (Trope and Liberman, 2003). In the same line, Conway and Loveday (2015) suggested that episodic nature of constructions related to the self are especially entailed in the access to recent memories and the simulation of near future events. Here we go a step further and put forward the idea that personal semantic representations are deeply involved in EFT by proposing a new neurocognitive model, which takes into account the role of PS as a function of the temporal distance in constructing episodic future thoughts (Figure 1). Accordingly, different types of future thoughts may exist as a function of temporal distance. In particular, we postulate (i) the existence of different mechanisms underlying subjective time travel as a function of temporal distance, i.e., increasing role of PS in the construction of EFT as a function of temporal distance, (ii) the shift from episodic to semantic forms of personal future representations as a function of temporal distance, and (iii) the role of self-relevance in maintaining EFT skills regardless of the temporal distance. As a result, we argue that episodic autobiographical representations are mostly involved in near future projections (i.e., next week) while personal semantic representations are mostly involved in distant future projections (i.e., in 5 years). It is worth pointing out that between near and distant future dimensions an intermediate temporal dimension exists (i.e., next year), which could be related to the progress of the role of PS in EFT and the shift from episodic to semantic forms of future thinking.

From a brain-based perspective, main results from the literature indicate a striking overlap between past and future episodic thinking that involves the medial prefrontal cortex (self-reference), the precuneus (visual mental imagery), and more especially the hippocampus (episodic binding process; Addis and Schacter, 2012; Dalla Barba and La Corte, 2013). However, some studies pointed out the additional activation of the inferior frontal and lateral temporal gyri, regions typically involved in semantic retrieval, in EFT (Viard et al., 2011). According to our view, we should observe a progressive decrease in hippocampal activity in distant forms of future thoughts and the increase activity in neocortical areas related to PS.

Nevertheless, even in distant future, it is possible to foresee unique events with an episodic connotation related to specific phenomenal features (sensorial, contextual, emotional), in which the hippocampal formation is involved, expectedly as far as the self-relevance and emotional connotation of imagined events is highly concerned.

## Clinical applications in neurodegenerative diseases and future directions

In conclusion, PS should play a crucial role in different temporal contexts within subjective time travel and semantic continuity. Our model delineates key elements of EFT that have been poorly highlighted in empirical studies such as temporal distance in episodic future thoughts and the specific role of the PS in personal prospection. It opens the avenue to further research in EFT and more generally in autobiographical domains to answer specific questions such as: What experimental paradigms will dissociate different forms of personal future thinking? What is the specific role of the hippocampus in the different distances of EFT?

Moreover it could represent a relevant road map for future research in particular in AD and SD patients, who represent excellent pathological models to enlarge the study of the episodic/semantic dissociation to the future dimension. Different studies have shown impaired capacity in personal prospection in AD patients. In particular the most part of them have underlined striking similarities in tasks performance in past and future condition with significant deficits in the generation of internal and external details for retrieved and imagined events (Irish and Piolino, 2015). A consistent number of neuropsychological investigations have also pointed out an impaired EFT capacity in SD patients (Duval et al., 2012; Irish et al., 2012; Viard et al., 2014).

While the most part of studies focused on the role of general semantic knowledge in constructing EFT in SD, Duval et al. (2012) investigate both structural (i.e., the episodic/semantic nature of self-representation) and functional features (i.e., consciousness, self-evaluation) dimensions of the self in a group of SD patients. These finding showed that SD patients were affected regardless of the episodic and semantic nature of self-representation in the future. Concerning the functional aspects of self, level of consciousness and self-projection were only impaired in the future. However, within the framework of this literature there is a notable variability in temporal distances used to value personal prospection abilities. Indeed, the great majority of studies employ a temporal dimension of 1 year to study EFT (Irish et al., 2012; Viard et al., 2014), on the other hand some studies have investigated the future thinking ability regardless of when the event will occur (De Vito et al., 2012; El Haj et al., 2015). Nevertheless, personal projection is a complex phenomenon and its investigation should take into account different temporal distances, which underlie different cognitive mechanisms involved in EFT. Specifically it is necessary to disentangle cognitive process involved in near vs. distant context of future in neurodegenerative diseases. Our model postulates that there is a double dissociation as a function of temporal distance given the increasing role of PS with temporal distance: AD patients will be more impaired in near EFT, which is mostly based on phenomenological continuity, whereas in SD patients a deep impairment in semantic continuity will affect mostly distant future thoughts.

## Concluding remarks: personal semantics and temporality

In line with the growing literature in EFT we propose a new neurocognitive model in which the role of PS in constructing EFT is a function of temporal distance in subjective mental travel in the future dimension. Namely the involvement of PS should increase in future mental time travel with distance of temporal context based on phenomenological continuity and semantic continuity.

## Author contributions

VLC and PP conceptualized the model and wrote the paper. VLC and PP approved the final version of the manuscript.

### Conflict of interest statement

The authors declare that the research was conducted in the absence of any commercial or financial relationships that could be construed as a potential conflict of interest.
